# Single-cell transcriptomics of human gut T cells identifies cytotoxic CD4^+^CD8A^+^ T cells related to mouse CD4 cytotoxic T cells

**DOI:** 10.3389/fimmu.2022.977117

**Published:** 2022-10-24

**Authors:** Shun Tanemoto, Tomohisa Sujino, Kentaro Miyamoto, Jonathan Moody, Yusuke Yoshimatsu, Yoshinari Ando, Ikuko Koya, Yosuke Harada, Anna Okuzawa Tojo, Keiko Ono, Yukie Hayashi, Kaoru Takabayashi, Koji Okabayashi, Toshiaki Teratani, Yohei Mikami, Nobuhiro Nakamoto, Naoki Hosoe, Haruhiko Ogata, Chung-Chau Hon, Jay W. Shin, Takanori Kanai

**Affiliations:** ^1^ Division of Gastroenterology and Hepatology, Department of Internal Medicine, Keio University School of Medicine, Tokyo, Japan; ^2^ Center for Diagnostic and Therapeutic Endoscopy , Keio University School of Medicine, Tokyo, Japan; ^3^ Research Laboratory, Miyarisan Pharmaceutical Co., Ltd., Tokyo, Japan; ^4^ RIKEN Center for Integrative Medical Sciences, Laboratory for Genomic Information Analysis, Yokohama, Japan; ^5^ Department of Surgery, Keio University School of Medicine, Tokyo, Japan; ^6^ Laboratory of Regulatory Genomics, Genome Institute of Singapore, Agency for Science Technology and Research (A*STAR), Singapore, Singapore

**Keywords:** cytotoxic CD4 T cells, single-cell RNA sequencing, human CD4^+^CD8A^+^ T cells, inflammatory bowel disease, intestinal inflammation

## Abstract

Cytotoxic CD4^+^ T cells (CD4-CTLs) show the presence of cytolytic granules, which include the enzymes granzyme and perforin. The cells have a pathogenic and protective role in various diseases, including cancer, viral infection, and autoimmune disease. In mice, cytotoxic CD4^+^ T cells express CD8αα^+^ and reside in the intestine (mouse CD4^+^CTLs; mCD4-CTLs). The population of cytotoxic CD4^+^ T cells in the human intestine is currently unknown. Moreover, it is unclear how cytotoxic CD4 T cells change in patients with inflammatory bowel disease (IBD). Here, we aimed to identify cytotoxic CD4^+^ T cells in the human intestine and analyze the characteristics of the population in patients with IBD using single-cell RNA-seq (scRNA-seq). In CD4^+^ T cells, granzyme and perforin expression was high in humanMAIT (hMAIT) cells and hCD4^+^CD8A^+^ T cell cluster. Both CD4 and CD8A were expressed in hTreg, hMAIT, and hCD4^+^CD8A^+^ T cell clusters. Next we performed fast gene set enrichment analysis to identify cell populations that showed homology to mCD4CTLs. The analysis identified the hCD4^+^CD8A^+^ T cell cluster (hCTL-like population; hCD4-CTL) similar to mouse CTLs. The percentage of CD4^+^CD8A^+^ T cells among the total CD4^+^ T cells in the inflamed intestine of the patients with Crohn’s disease was significantly reduced compared with that in the noninflamed intestine of the patients. In summary, we identified cytotoxic CD4^+^CD8^+^ T cells in the small intestine of humans. The integration of the mouse and human sc-RNA-seq data analysis highlight an approach to identify human cell populations related to mouse cell populations, which may help determine the functional properties of several human cell populations in mice.

## Introduction

Cytotoxic CD4 T cells (CD4-CTLs) contain cytolytic granules that release enzymes such as granzymes(GZM) and perforin(PRF). CD4-CTLs have been identified in patients with chronic viral infections, cytomegalovirus, EB virus, hepatitis B and C, and acute influenza viral infections (1–8). Recently it has been shown that COVID-19 vaccine-induced CD4-CTLs (9) recognize viral antigens in a major histocompatibility complex (MHC) class II dependent manner (10). Moreover, CD4-CTLs can target other infected cells, such as epithelial and B cells. In murine models, CD4-CTLs are characterized by the expression of CD4^+^ and CD8α^+^, production of GZM, and PRF and are located in the intraepithelial compartment (CD4^+^CD8α^+^ intestinal epithelial lymphocytes (IELs)) (11–15). CD4^+^CD8α^+^ IELs are known to increase by administration of a diet rich in L-tryptophan and the prevalence of the specific aryl hydrocarbon receptor-L-producing microbe, *Lactobacillus reuteri* (16). Studies indicate that CD4^+^CD8α^+^ IELs protect against autoimmune colitis in a food allergy model (15, 17), and exacerbated colitis is observed on re-encountering the cognate or bacterial antigen (11, 18); however, their precise role in the intestine is unclear.

In humans, CD4^+^CD8A^+^ T cells are detected in many organs, such as the blood of supercentenarians (19, 20)and the skin of patients with systemic sclerosis (21). In the human intestine, CD4^+^CD8A^+^ T cells recognize microbes, especially *Faecalibacterium prausnitzii*, and produce IL-10 (22). Human CD4-CTLs are enriched in CD3^+^CD4^+^CD45RA^+^CCR7^-^ T cells in the blood (23). A previous study performed single cell RNA sequencing (sc-RNA-seq) using samples from patients with ulcerative colitis (UC) and Crohn’s disease (CD) to identify the effector T cell population. The study demonstrated that the T-helper 17 (Th17) and T-helper 1 (Th1) were expanded in the active patients with UC and Crohn’s disease (24). Accumulating evidence shows that cytokine-producing CD4^+^ T cells (Th1 and Th17 cells) induce inflammation in the gut (25). However, the connection between CD4^+^CD8A^+^ T cells and cytotoxic gene expression in the human intestine and the CD4-CTL markers in the gut disease, including IBD have not been investigated. Therefore, we aim to annotate cell populations that express cytotoxic genes in CD4^+^ T cells and evaluate their CD8 expression using scRNA-seq.

The evaluation of immune cells in humans and mice is challenging because their levels and gene expression show differences in the two systems. For instance, TRAV1-2 and TRAJ33 can identify MAIT cells in humans and mice (26–29). However, MAIT cells are the major population of immune cells in humans, they are rare in the mice (30–33). A few immune cells are recognized as belonging to the same population based on their gene expression profiles. For instance, mouse regulatory T cell (Tregs) in peripheral tissue express *Batf*, *amphiregulin*, *IL-10*, and *ST2* with *Foxp3*, whereas human tissue repair Tregs express *Batf* and *CCR8* (34–39). These populations were defined using single-cell chromatin accessibility assays and a shared tissue repair Treg signature on comparing mouse and human tissue-resident Tregs. Considering these findings, we hypothesized that the integration of the gene signatures of the murine and human intestinal T cells can identify the human cluster related to mouse CD4-CTLs.

To test this hypothesis, we aimed to identify cytotoxic CD4^+^ T cells in the human intestine and characterize the populations in patients with IBD. We performed scRNA-seq of the small intestinal samples of patients with IBD and mice and integrated the dataset using fast gene set enrichment analysis (fGSEA). We identified CD4^+^ T cells, which expressed CD8A^+^ in human Treg (hTreg) cluster and the hMAIT cluster, which expressed cytotoxic genes in intestine. We also performed differential expression analysis and uncovered CD27 is the potential surface marker to divide hTreg and hMAIT/hCD4^+^CD8A^+^ cluster. Finally, we compared the CD8A^+^ T cells among CD4^+^ T cells in patients with IBD, especially CD. The proportion of CD27^-^CD4^+^CD8A^+^ T cells in inflamed tissue of patients with CD was less compared to the noninflamed tissues in the same patients. We identified hCD4^+^CD8A^+^ cluster as a novel human CD4-CTL population related mCD4-CTLs using sc-RNA-seq and fGESA analysis after integration with mouse scRNA-seq data, which will enable the analysis of the difficult to assess novel human cell population in future studies.

## Methods

### Animals

Female C57BL/6J (WT) mice (8–11 weeks old) were purchased from Japan CLEA (Tokyo, Japan). All mice were maintained under specific pathogen-free conditions in the Animal Care Facility of Keio University School of Medicine. All experiments were approved by the Regional Animal Study Committees (Keio University, Tokyo, Japan) and were performed according to Institutional Guidelines and Home Office Regulations.

### Human samples

Small intestinal tissue samples were obtained from nine patients with colon cancer (controls), one with UC, and eight with CD who underwent surgical resection. The characteristics of these patients are shown in **Table 1**. UC and CD were diagnosed by the criteria defined by the research group of IBD at the Ministry of Health, Labor and Welfare in Japan. The intestinal tissue samples from the controls were obtained from patients with colon cancer who underwent ileum-colon resection.

**Table 1:** Patient characteristic.

	No		Age	M/F	Location	Clinical stage	Operation
control	1	colon cancer	48	F	AC	T3N0M0	ilio-cecal sectomy
	5	colon cancer	85	F	AC	T3N1M0	ilio-cecal sectomy
	7	colon cancer	42	M	AC	T1N0M0	ilio-cecal sectomy
	9	colon cancer	60	F	AC	T3N0M0	ilio-cecal sectomy
	11	colon cancer	80	M	AC	T1N0M0	ilio-cecal sectomy
	22	colon cancer	63	M	AC	T3N0M0	ilio-cecal sectomy
	23	colon cancer	67	F	AC	T4N0M0	ilio-cecal sectomy
	52	colon cancer	68	F	AC	T2N1M0	ilio-cecal sectomy
	54	colon cancer	81	M	AC	T3N2M0	ilio-cecal sectomy
	**No**	**CD/UC**	**Age**	**M/F**	**Disease status/active**	**Disease Type**	**Operation**
IBD	6	CD	54	F	stenosis. Active disease	intestinal type	ileum resection
	7	CD	25	F	active ulcer	intestinal type	ileum resection
	8	CD	40	M	stenosis. Active disease	colon-intestinal	ileum resection
	15	CD	40	M	stenosis. Active disease	colon intestinal	ileum resection
	17	CD	37	F	stenosis. Active disease	colon intestinal	ileum resection
	22	CD	29	M	stenosis. Active disease	colon intestinal	ileum resection
	23	CD	53	M	stenosis. Active disease	colon intestinal	ileum resection
	9	UC	27	M	UC active	pan colitis	colon resection

AC, Ascending colon.

Single cell analysis: HD-No.52,54

Single cell analysis: IBD-No.7,9

### Ethics

All experiments were approved (20170255) by the Institutional Review Board of Keio University School of Medicine, and written informed consent was obtained from all patients in accordance with the Declaration of Helsinki.

### Isolation of intestinal epithelial lymphocytes (IELs) and lamina propria (LP) mononuclear cells from mice and human samples

A mouse was euthanized by cervical dislocation and the colon was collected without mesentery. The colon was opened longitudinally and washed with Ca^2+^, Mg^2+^-free Hank’s balanced salt solution (HBSS) (Nacalai Tesque, 17460-15) to remove fecal content. After washing, the colon was further cut into small pieces and incubated with HBSS containing 1 mM dithiothreitol (Invitrogen, 15508-013) and 5 mM EDTA (Thermo Fisher Scientific, 15575038) for 30 min at 37°C to remove the epithelial layer. After removal of epithelial layer, the mucosal pieces were washed with HBSS and dissolved into solutions by incubation with HBSS containing 1.5% fetal bovine serum (FBS) (Thermo Fisher Scientific, 10270-106), 1.0–3.0 mg/mL collagenase (Wako, 032-22364), and 0.1 mg/mL DNase (Sigma-Aldrich, DN25-1G) for 45min at 37°C. The dissolved solution was centrifuged at 600 x *g* for 5 min, and the pellet was resuspended in 40% Percoll (GE healthcare, 17-0891-01) and overlaid on 75% Percoll. Percoll gradient separation was performed by centrifugation at 800 x *g* for 20 min at 20°C. LPMCs were collected at the interphase between 40% and 75% Percoll layers. As for colonic epithelial cells, the supernatant containing removed epithelial layer as described above was collected and centrifuged at 600 x g for 5 min at 4°C and the pellet was resuspended in 40% Percoll. Percoll gradient separation was performed as well. Epithelial cells, existing in the upper layer, were collected, washed, and resuspended with appropriate buffer. The numbers of live cells were determined by Countess II (Thermo Fisher Scientific) (17). Human samples were obtained from the resected intestine of controls and patients with UC and Chrons disease. In controls, the tissue samples were collected far from the cancer lesions. The human samples were processed similarly to those of mice to collect IELs and LPs.

### Preparation of lymphocytes from mice

Mesenteric lymph nodes were also harvested as well. Fat tissues around these lymph nodes were carefully removed and the lymph nodes were homogenized manually in HBSS and the lysates were filtered through 100-µm cell strainer, and the passed cells were collected for analysis. Spleen was harvested from mice after euthanized and homogenized manually in HBSS. The lysates were filtered through 100-µm cell strainer. The passed cells were hemolyzed with 0.84% (v/w) ammonium chloride (Nacalai, 02424-55), washed with HBSS and collected for analysis after the solution was centrifuged at 600 × *g* at 4°C for 5 min.

### Fluorescence-activated cell sorting (FACS) analysis

The surface antigens of isolated single cell suspensions were pre-incubated with an FcγR-blocking mAb (anti-mouse CD16/32 (2.4G2), BD Biosciences, 553142) for 20 min before staining cell surface antigens. After FcγR-blocking, fluorescence-conjugated specific mAbs to surface molecules were incubated at 4°C protected from light for 30 min. After staining surface molecules, the cells were washed and resuspended with PBS containing 0.5% (w/v) bovine serum albumin (BSA) (Nacalai, 01863-48). The prepared cells were incubated with the specific fluorescence-labeled monoclonal antibodies (mAbs) at 4°C for 30 min, using an intracellular staining kit (ebioscience, 00-5523-00). FITC anti-CD4 (RM4-5, 553047), PerCP-Cy5.5 anti-CD4 (RM4-5, 56115), PE anti-CD8α (53-6.7, 553032) and Brilliant Violet (BV)510 anti-CD45 (30-F11, 563891) were purchased from BD Biosciences. APC anti-Foxp3 (FJK-16s, 17-5773-80) and PE-Cy7 anti-CD8β, (eBioH35-17.2, 25-0083-82) were purchased from eBioscience. For human sample analysis: PerCP-Cy5.5 anti-human CD4 (BD Biosciences, RMA4-5, 357413), PE anti-human CD8A (BD Biosciences, OKT8,568398) BV421 anti-human CD8B (BD Biosciences, 2ST8.5H7, 742390), FITC-human TCRB (BD Biosciences, T10B9.1A-31, 555547), APC anti-human CD27 (Bio Legend, LG.3A10, 124211) were used. Dead Cells were excluded using a Fixable Viability Dye eFluor (eBioscience, 65-0865-14). Events were acquired with FACS Canto II (BD Biosciences), sorted using BD FACS Aria-II (BD Bioscience), and analyzed using FlowJo software (BD Biosciences) (17).

### Single-cell RNA sequencing (Sc-RNA seq)

#### Mouse Sc-RNA seq

CD4^+^CD45^+^ cells were obtained from the pooled Small Intestine(SI)-IntraEpithelial and SI-LaminaPropria mononuclear cells of four mice (8,000 cells each) and loaded into Chromium Controller (10X Genomics). RNA-seq libraries were prepared using a Chromium single-cell 5’ gene expression library preparation reagent kit (5’ v1.1, mouse) according to the manufacturer’s instructions (10X Genomics). The libraries were then sequenced using a DNBSEQ (MGI Tech). Sequence reads from all samples were processed by Cell Ranger (10X Genomics). Seurat (v 4.04) (40) was used to aggregate and analyze the processed data following the official vignettes (https://satijalab.org/seurat/articles/integration_introduction.html). Specifically, PCA analysis was performed to identify clusters, and 10 gene clusters (1–10) were projected on Uniform Manifold Approximation and Projection (UMAP) space with the normalized gene expression. The gene expression heatmaps showing the unbiased generation of the top 10 differentially expressed genes for each cluster were constructed.

#### Human Sc-RNA seq

TCRB^+^ cells were obtained from the pooled SI-LP mononuclear cells of each patient (10,000 cells each) and loaded into Chromium Controller (10X Genomics). RNA-seq libraries were prepared using Chromium single-cell 5’ gene expression library preparation (human) according to the manufacturer’s instructions (10X Genomics). The libraries were then sequenced using a DNBSEQ (MGI Tech). Sequence reads from all samples were processed by Cell Ranger (10X Genomics). Seurat (v 4.04) (40) was used to aggregate and analyze the processed data by following the official vignettes (https://satijalab.org/seurat/articles/integration_introduction.html). Specifically, PCA analysis was performed to identify clusters, and 18 gene clusters (1–18) were projected on UMAP space with the normalized gene expression. Gene expression heatmaps showing the unbiased generation of the top 4 differentially expressed genes for each cluster were developed. Gene ontogeny analysis was performed using clusterProfiler (v 4.0.5) (41).

### fGSEA analysis of 100 top genes

Gene sets were created for each of the 10 mouse single-cell clusters using the top 100 genes identified by Seurat FindMarkers with default parameters that could be mapped to human gene names using biomaRt. For each of the 18 human single-cell clusters, a ranked gene list was created using Seurat FindMarkers avg_logFC. fGSEA was used to calculate the enrichment of mouse-cluster gene-sets within each human-cluster ranked gene list using the parameters (eps= 0.0, minSize = 15, maxSize = 500 with ‘signedP’ corresponding to the sign of the fold change multiplied by -log10 (pvalue).

### Correlation of the published scRNA-seq data to our scRNA-seq data

Transferring the “labels” from a published dataset to our dataset using Celltypist algorithm (https://www.celltypist.org). Briefly, A celltypist model was trained to predict the Joint_Cluster label using log normalized expression of all genes from a previous scRNA-seq dataset (Nat.Med. https://doi.org/10.1038/s41591-020-1003-4) using default parameters (42–44).

### Statistical analyses

All values are shown as mean ± SEM. Statistical analyses were performed using GraphPad Prism 8 (GraphPad Software). To compare data of two groups, statistical differences were evaluated using the two-tailed unpaired Student’s t-test (for normally distributed variables). Differences between more than three groups were tested using one-way ANOVA followed by Tukey’s multiple comparisons test (for normally distributed variables). Two-way repeated ANOVA followed by Bonferroni’s multiple comparisons test was used if needed.

### Data deposit

All the single RNAseq data deposit at DDBJ-DRA014019 (mouse) and GSE210955 (human).

## Results

### Mouse CD4-CTLs expressing CD8a were composed of a single population

**Figure 1:**
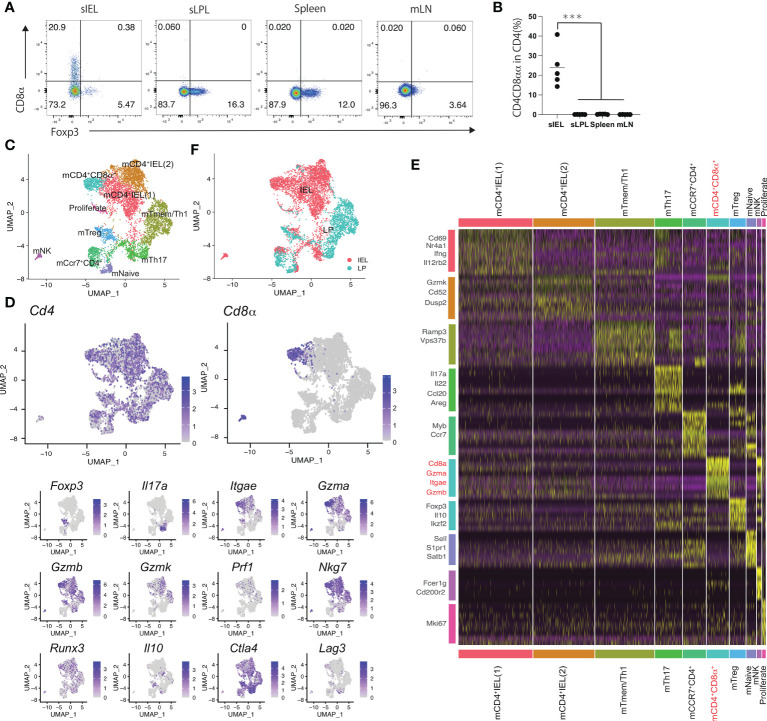
Mouse CD4 cytotoxic T cells expressing CD8a are composed of a single population **(A)** Eight-week-old C57BL/6J mice received the CE2-diet (Ctrl-diet; n=5/group). Representative flow cytometry plots for Foxp3 expression in CD4 positive lymphocytes in the small intestine intraepithelial lesion (sIEL), lamina propria (sLPL), spleen, and mesenteric lymph node (mLN). **(B)** Percentage of CD8α^+^ cells among the CD4^+^TCRβ^+^ lymphocyte subset in sIEL, sLPL, spleen, and mLN. **(C)** Single-cell analysis was performed using CD4^+^ T cells in IEL and LPL from 11weeks-old WT mouse. All cells were classified into clusters (1–10) and analyzed using uniform manifold approximation and projection (UMAP). **(D)** Heat map for gene expression, for genes expressed in mCD4-CTLs (*Cd4*, *Cd8α*, *Gzma*, *Gzmb*, *Gzmk*, *Itgae*, *Prf1, Nkg7*, *Runx3*) and helper T cells (*Foxp3*, *Il17a, Il10, Ctla4, Lag3*). **(E)** Genes from each cluster (1–10) using data from the single-cell analysis. mCD4^+^CD8α^+^ cluster and related genes are highlighted **(F)** Cells were colored in red (IEL) and blue (LPL). *P*-values were obtained using the Student’s *t*-test. ***<0.001. Data are shown as the mean ± SEM **(B)** or ± SD **(C)** from individual mice (n = 5 for A) and are representative of two independent experiments.

CD4^+^ T cells in mice expressing granzyme and perforin express CD8αα (17). In this study, the analysis of mouse CD4^+^CD8αα^+^ T cells in each organ revealed that CD4^+^CD8αα^+^ T cells were rarely detected in the spleen, mesenteric lymph nodes (mLNs), and LP of the small intestine but were f detected in IELs (**Figures 1A, B**). Therefore, we hypothesized cytotoxic CD4^+^CD8αα^+^ T cells exist as a single population in the intestinal tract. To test this, we performed sc-RNA-seq for CD4^+^ T cells in the LP and intraepithelial compartment (IE), as CD4^+^ CD8αα ^+^ intraepithelial cells develop from CD4^+^ T cells in the LP. A total of 6146 cells were analyzed and assigned to 10 clusters (1–10) based on similarities in gene expression.

The identified clusters were named based on the localization and feature genes (**Figures 1C–E**). Most CD4^+^ T cells in mouse (m) CD4_IEL_ (1), mCD4_IEL_ (2), and mCD4^+^CD8α^+^ clusters comprised the IEL population, and CD4^+^ T cells in the mTmem/Th1 cluster were from the LP lymphocytes (LPL) population. The remaining clusters included a mixture of IEL and LPL cells (**Figure 1C**). Cells in all cluster express *Cd4*. We exclude the NK cells cluster with the high expression of *Cd200r2*, *Fcer1g*. mCD4_IEL_ (1) cluster expressed *Cd69*, *Ifng*, and *Il12rb2*, which include the memory IEL cells that are the precursor of mCD4-CTL. *Nr4a1* expressed highly in m mCD4_IEL_ (1) cluster. In the remaining major IEL population, mCD4_IEL_ (2) cluster was characterized by genes including *Ccl5*, *Gzmk*, and *Cd52*. mTmem/Th1 was annotated as the memory T cell population and included *Vps37b* and *Ramp3*. *Il17a*, *Il22, Ccl20*, and *Areg* were enriched in the mTh17 cluster. *Foxp3, Il10*, and *Ikzf2* were present in mTreg cluster. mNaïve T cells were identified by *Sell*, *S1pr1*, and *Satb1*. Proliferate CD4 T cells were defined by the expression of *Mki67*. *Ccr7* and *Myb* expressed cells were annotated as mCcr7CD4 cluster. *Cd8a*, *Itgae*, and cytotoxic genes such as *Gzma* and *Gzmb* were expressed in the mCD4^+^CD8α^+^ cluster. In the UMAP analysis, *Cd8α* was expressed in the mCD4^+^CD8α^+^ cluster alone, whereas *Gzma*, *Gzmb*, and *Itgae* were expressed in mCD4_IEL_ (1), mCD4_IEL_ (2), mCD4^+^CD8α^+^, mTmem/Th1, and mTreg clusters (**Figure 1D**). *Runx3*, a master gene of the CD8 T cells was expressed in mCD4^+^CD8α^+^ clusters. *Foxp3* was expressed in the mTreg cluster, and *Il17a* was expressed in the mTh17 cluster. As granzyme expression was high in the mCD4^+^CD8α^+^ cluster, we named the mCD4^+^CD8α^+^ cluster mouse cytotoxic CD4^+^ T cells (mCD4-CTLs) population. Taken together, mCD4-CTLs expressing both CD4^+^ and CD8α^+^ were annotated as belonging to one cell cluster type in the mouse intestine.

### Single-cell analysis of samples from control tissues and patients with ibd identified 10 cell populations

**Figure 2:**
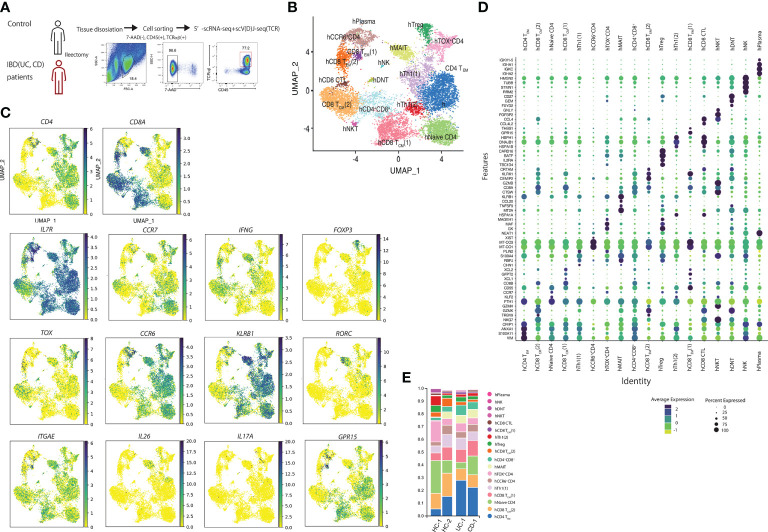
Human single-cell analysis in controls and patients with inflammatory bowel disease showed that cells are composed of 10 populations. **(A)** Sorting strategy for the human single-cell RNA-seq data: LPLs collected from digested small intestinal tissues were sorted by identifying 7AAD^–^CD45^+^TCRB^+^ cells by FACS. **(B)** Uniform manifold approximation and projection (UMAP) embedding of a total of 12,073 cells from controls (n=2) and patients with inflammatory bowel disease (IBD) (n=2) (7012 cells from controls, 531 cells from UC, and 4530 cells from CD patients). Cells were clustered into 18 clusters. **(C)** Expression of *CD4, CD8A, IL7R, CCR7*, *FOXP3*, *IFNG*, *Il17A, TOX, CCR6, KLRB1, RORC, ITGAE, IL26, IL17A* and *GPR15* in UMAP. **(D)** featuring genes in human T cell clusters. **(E)** Proportion of each cluster in controls and patients with IBD.

Next, we performed scRNA-seq of the samples from the human intestine to evaluate the CD4-CTLs in the human small intestine. TCRB^+^ T cells from two controls (small intestine apart from the colon cancer lesion), one patient with UC, and one with CD (both noninflamed lesions) were obtained (**Figure 2A**). A total of 12,073 cells in the gut (7012cells from controls, 531 cells from patients with UC, and 4530 cells from patients with CD) were analyzed based on their gene expression profiles and assigned to 18 clusters by their featuring gene (**Figures 2B–D**). We identified the small populations of hNK, hNKT, hDNT, and hPlasma based on gene expression data. Then, we focused on the other populations and visualized the CD4^+^ and CD8A^+^ expressing cells (**Figure 2D**). *IL7R* was expressed in most of the clusters. Human CD4^+^ T cells expressing *S100A11*, *VIM*, *ANXA*, and *CRIP1* comprised the human CD4 T effector memory (hCD4T_EM_) cluster. Naïve CD4 cells expressed *KLF2* and *CCR7*. *IFNG* was expressed high in hTh1 (1) and hTh1 (2) clusters. hTh1 (1) population featured as *DNABJ*, and hTh1 (2) population express *PLIN2* gene. *BATF*, *IL2R*, and *CARD16* were expressed in the hTreg cluster. Cells in the hTreg cluster expressed *FOXP3*. CD4 T cells cluster between hTregs and hTh1 (1) cluster was identified as ThOX^+^ cluster as *TOX* expression was high in CD4 T cells in this cluster. CD4 T cells expressing *CCR6* were annotated as hCCR6^+^ cluster. They also express *MT-CO1*. *CCL20*, *KLRB1*, *MT2A*, *RORC, ITGAE*, and *IL26* were expressed in hMAIT cells. *IL17* was expressed in the small population of hMAIT and hTreg cluster. In *CD8A* positive cells, we defined T_EM_ and T_CM_ by the expression of *CCR7*. Two populations were identified in CD8T_EM._ *GPR15* was highly expressed in hCD8T_EM_ (1) and *GZMK* in hCD8T_EM_ (2). Cells in hCD8 T_CM_ (1) cluster highly expressed *XCL1* and *XCL2*, whereas cells hCD8T_CM_ (2) cluster expressed *CCL4* and *GZMK*. hCD8CTL population was identified between hCD8T_CM_ (2) and hCD8T_EM_ (2) and expressed *CRTAM*, *GZMB*, and *GZMK.* Moreover, cell population, which was between CD4 Th1 (2), CD8T_CM_ (1), and CD8T_CM_ (2), was identified as the hCD4^+^CD8A^+^ cluster.

We then evaluated the difference in the proportion of cells in each cluster between the controls and patients with IBD (**Figure 2E**). The proportion of hCD4T_EM_ in patients with IBD was higher than in controls. These data are consistent with the previous findings that CD4 effector cells were important for the pathogenesis of IBD. However, we did not observe other specific cell populations between patients with IBD and controls.

### Cytotoxic gene expression in human CD4^+^CD8A^+^ T cells

**Figure 3:**
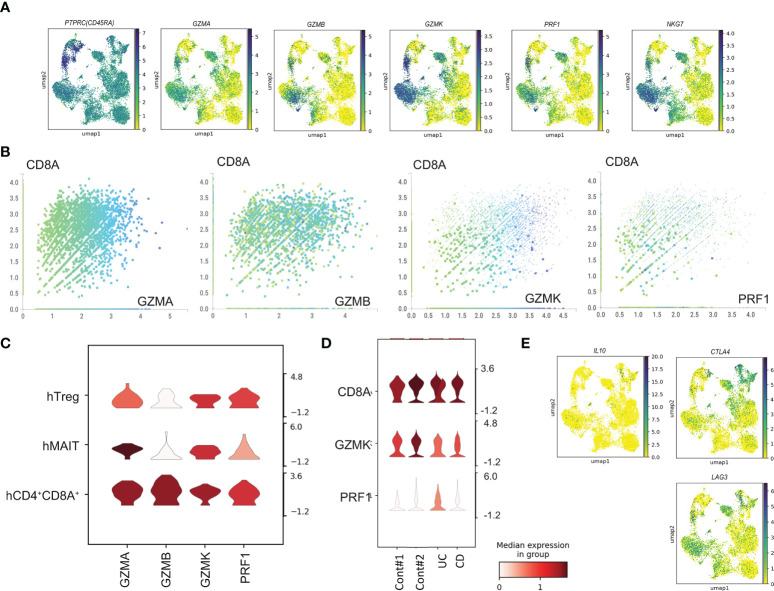
Cytotoxic gene expression in human T cells from sc-RNA-seq data. **(A)** Gene expression of *PTPRC, GZMA, GZMB, GZMK, PRF1* and *NKG7* in uniform manifold approximation and projection (UMAP) plots using human sc-RNA-seq data. **(B)** The expression level of CD8A and GZMA, GZMB, GZMK, PRF1 in each cell. **(C)** The expression level of *GZMA, GZMB, GZMK, PRF1* in hTreg, hMAIT, and hCD4^+^CD8A^+^ clusters. **(D)** The expression level of *CD8A, GZMK*, and *PRF1* of CD4+CD8A+ T cells from control, UC and CD patient. **(E)** Gene expression of *IL10, CTLA4*, and *LAG3* in uniform manifold approximation and projection (UMAP) plots using human sc-RNA-seq data.

Next, we evaluated the expression of cytotoxic marker genes (**Figure 3A**). CD4-CTLs in the intestine expressed CD45RA. Cytotoxic makers, GZMA, GZMB, GZMK, PRF1 and NKG7 were expressed in hMAIT, hCD8 T_CM_ (2), hCD8 T_EM_ (2), hCD8CTL, and hCD4^+^CD8A^+^ populations. Most cells were annotated to the CD8α^+^ population, and the comparison of the expression of the cytotoxic marker genes with *CD8A* expression (**Figure 3B**) revealed a positive correlation with *CD8A* expression. Furthermore, analysis of the cytotoxic gene expression in the CD4^+^ T cells demonstrated high expression of *GZMA* in the hMAIT cluster and *GZMA, GZMK*, and *PRF* in the hCD4^+^CD8A^+^ cluster (**Figure 3C**). Taken together, human CD4-CTLs were in hMAIT, hTreg, and hCD4^+^CD8A^+^ clusters. We then analyzed the expression level of cytotoxic genes in CD4^+^CD8A^+^ T cells from controls and patients (**Figure 3D**). Cytotoxic gene expression in CD4^+^CD8A^+^ T cells was comparable between control and IBD. These data suggest that the capacity of cytotoxic gene expression is unchanged between control and IBD patients. CD4^+^CD8A^+^ T cells in the intestine expressed *IL10*, which is essential for the inhibition of colitis (27). The expression analysis of *IL10, LAG3*, and *CTLA4* revealed *IL10* in hTregs (**Figure 3E**). Other inhibitory genes, *LAG3* and *CLTA4*, were expressed hTreg, hMAIT, hCD8T_CM_, and hCD4^+^CD8A^+^ populations. These data indicated the presence of human CD4-CTLs in hTreg, hMAIT, and hCD4^+^CD8A^+^ clusters, and all these clusters expressed CD8A.

### Comparison of CD4+CD8A+ T cells between our data and public dataset

**Figure 4:**
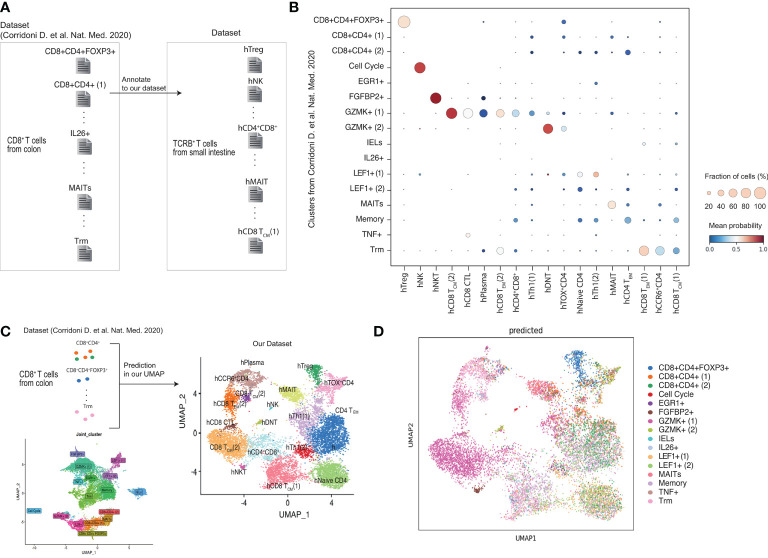
Comparison of CD4+CD8A+ T cells between our data and published data set. **(A, B)** Obtained scRNA-seq data set from previous paper (Corrinoni D. et al) and annotate to our clusters. **(C, D)** Prediction the cells obtained in the previous report belong to our cluster.

Previous scRNA-seq studies reported the CD8A^+^CD4^+^ T cells in human colon (45). Corridoni et al (45). sorted CD8^+^T cells in patients with UC and identified populations expressing CD4. A comparison of our study with Corridoni et al.’s revealed that the CD8^+^CD4^+^FOXP3^+^ cluster identified in Corridoni et al.’s study was highly correlated with the hTregs cluster identified in our study (**Figures 4A, B**). However, the CD8^+^CD4^+^, EGR1^+^, IELs, IL26^+^, and LEF1^+^ clusters in Corridoni et al.’s study did not match the clusters identified in our study. FGFBP2^+^ of Corridoni et al.’s was annotated to the hNKT cluster. The GZMK^+^ cluster in Corridoni et al.’s study was associated with the hCD8T_CM_, hCD8CTL, hCD8T_EM_, and CD4^+^CD8^+^ clusters in our study. The MAITs and Trm clusters in Corridoni et al.’s study matched the hMAIT and hCD8T_EM_ (1) clusters in our study, respectively. These data suggest that CD8^+^CD4^+^FOXP3^+^ cells and MAITs identified in Corridoni’s data set are highly related to hTregs and hMAIT in our data set, respectively, but the CD4^+^CD8^+^ cluster from Corridoni’s data set did not match hCD4^+^CD8^+^ cluster in our data set.

Furthermore, we compared our UMAP data with previously reported cell population data (**Figures 4C, D**). The analysis identified CD8^+^CD4^+^FOXP3^+^cells clustered in hTreg and MAITs in hMAIT. However, the CD8^+^CD4^+^ T cells from Corridoni et al.’s data did not match our UMAP data. Moreover, cells in GZMK^+^ (1) from Corridoni et al.’s data set were located in the hCD4^+^CD8^+^ cluster of our UMAP.

### Cells in hCD4^+^CD8A^+^ cluster is close to mCD4-CTLs by fGSEA comparing mouse and human scRNAseq

**Figure 5:**
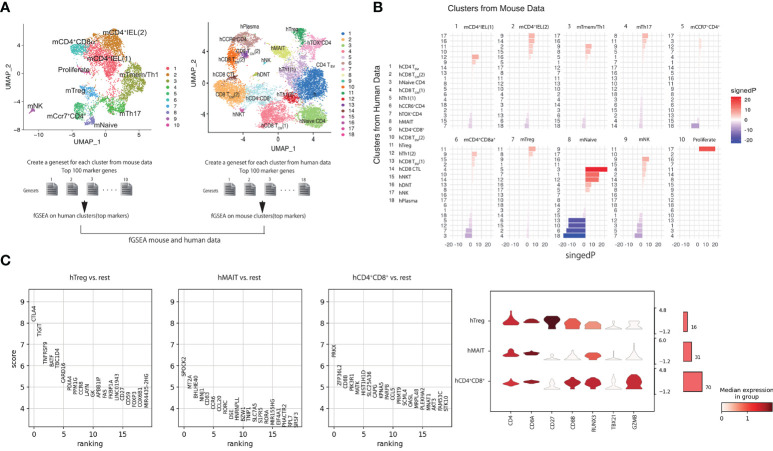
Cells in hCD4^+^CD8A^+^ cluster are similar to mCD4-CTLs by using fGSEA**(A)** Schema of the integrated mouse single-cell RNA-seq data with the human single-cell RNA-seq data using fGSEA. **(B)** Enrichment of mouse cluster gene sets within each human gene cluster *via* gene set enrichment analysis. **(C)** Top 20 genes in CD4^+^CD8A^+^ T cells expressed in hTreg, hMAIT, and hCD4^+^CD8A^+^ clusters compared with other cell populations. **(D)** Expression of CD4, CD8A, CD27, CD8B, RUNX3, TBX21, and GZMB in CD4^+^CD8A^+^ T cells in hTreg, hMAIT, and hCD4^+^CD8A^+^ clusters.

We noticed the CD4-CTLs were in the three populations from our RNA-seq data. We further investigated which human T cell population was functionally similar to the mCD4 T cell population, especially mCD4-CTLs (**Figure 5A**). We analyzed the top 100 marker genes in each mouse cluster for fast GSEA and constructed a heatmap of each cluster from humans and mice to identify highly similar (shown in red) and non-related (shown in blue) populations (**Figure 5B**). mNaive T cells were highly correlated to hNaive CD4 T cells as expected, and mTregs cluster was highly correlated with hTregs. mCD4^+^IEL (1) cluster showed high correlation with hTh1 and hCD4^+^CD8A^+^ clusters, whereas mCD4+IEL (2) cluster was highly correlated with hCD4^+^CD8A^+^ and hCD8 CTL cluster. In addition, mTmem/Th1 cluster was close to hMAIT and hCD4^+^CD8A^+^ clusters. The mTh17 and mCD4^+^CD8A^+^ clusters were close to hMAIT and hCD4^+^CD8A^+^ clusters. Interpreting the fGSEA results, we assigned hCD4^+^CD8A^+^ cluster as being functionally associated with mCD4-CTLs, rather than hTreg and hMAIT cluster.

Previous studies have shown that CD4-CTLs in human blood express CD45RA; however, all cells in intestine expressed CD45RA (**Figure 3A**). To identify the specific surface marker of hCD4^+^CD8A^+^ cluster, we next analyzed differentially expressed genes in CD4^+^CD8A^+^ T cells in hTreg, hMAIT, and hCD4^+^CD8A^+^ clusters. The CD4^+^CD8A^+^ T cells in hTreg cluster showed high expression of *CLTA4*, *BATF*, *CCR8*, and *FOXP3*, also known as Treg marker genes. CD4^+^CD8A^+^ T cells in hMAIT cluster showed high expression of *CCR6*, *CCL20*, and *RORC*, also known as Th17 marker genes. Additionally, CD4^+^CD8A^+^ T cells in h CD4^+^CD8A^+^ cluster expressed *CD8B* and *CCL5*. We mapped the expression level of *CD4*, *CD8A*, *CD8B*, and *CD27* in CD4^+^CD8A^+^ T cells of each cluster with *RUNX3*, *TBX21*, and *GZMB*, which correspond to *Runx3*, *Tbx21* and *Gzmb* in m-CD4CTLs (**Figure 4B**). CD27 was highly expressed in hTreg but showed reduced expression in hMAIT and hCD4^+^CD8A^+^ clusters. *CD8B* was expressed in hCD4^+^CD8A^+^ cluster, showed modest expression in hTreg and hMAIT clusters. The genes *RUNX3* and *GZMB* were expressed in hCD4^+^CD8A^+^ cluster as determined previously using fGESA with mouse and human sc-RNA-seq data. *TBX21* showed higher expression in hCD4^+^CD8A^+^ cluster than other 2 populations. Taken together, CD8B and CD27 could be the surface marker to distinguish CD4^+^CD8A^+^ T cells in each cluster.

### The proportion of CD4^+^CD8A^+^ T cells in the inflamed intestine is reduced compared to that in the noninflamed intestine of patients with CD

**Figure 6:**
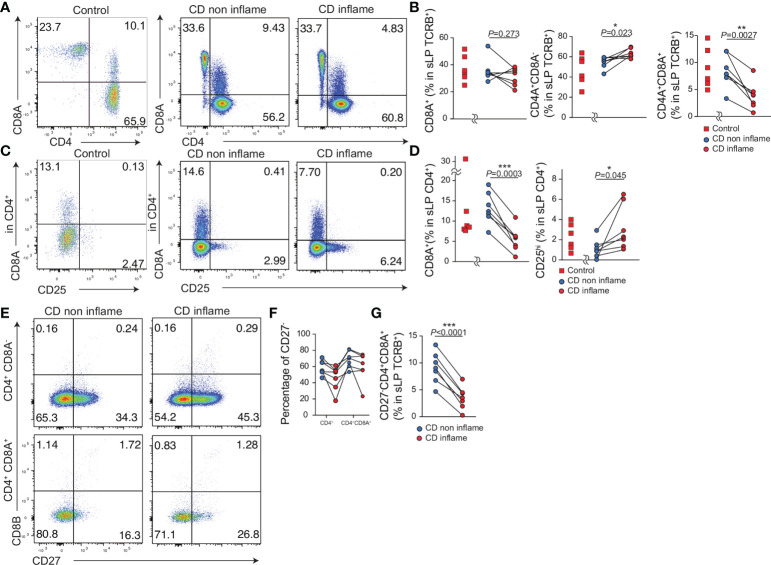
The proportion of CD4^+^CD8A^+^ T cells in the inflamed intestine is reduced compared with the non-inflamed intestine in patients with CD. Intestinal lymphocytes were obtained from the resected small intestine of controls (n=7) and patients with CD (n=7). Inflamed and non-inflamed tissues were obtained from the same CD patients (CD non inflame, CD inflame respectively). **(A)** Representative flow cytometry plots of CD4 and CD8A expression by TCRB positive lymphocytes in the small intestine lamina propria (sLPL). **(B)** Percentage of CD8A^+^, CD4^+^CD8A^–^, and CD4^+^CD8A^+^ cells among the TCRB^+^ lymphocyte subsets in sLPL. **(C)** Representative flow cytometry plots for CD25 and CD8A expression in CD4^+^TCRB^+^ T lymphocytes in sLPL. **(D)** Percentage of CD25^+^ and CD8A^+^ cells among the CD4^+^TCRB^+^ lymphocyte subset in sLPL. **(E)** Representative flow cytometry plots for CD27 and CD8B expression in CD4^+^CD8A^–^TCRB^+^ and CD4^+^CD8A^+^TCRB^+^ T lymphocytes in sLPL. **(F)** Percentage of CD27^–^ cells among CD4^+^CD8A^–^TCRB^+^ and CD4^+^CD8A^+^TCRB^+^ T lymphocytes in sLPL. **(G)** Percentage of CD27^–^CD4^+^CD8A^-^ cells among the TCRB^+^ T lymphocytes in sLPL. *P*-values were obtained *via* the paired Student’s *t*-test. *<0.05, **<0.01, ***<0.001. Each dot represents data from an individual (n = 7).

Lastly, we evaluated whether the population of CD4^+^CD8A^+^ T cells was altered in the inflamed or noninflamed intestinal tissue in CD patients. We obtained resected intestinal tissue from controls (N = 6) and patients with CD (N = 7) and analyzed the expression of CD4^+^ and CD8A^+^ in TCRB^+^ T cells comparing inflamed and noninflamed intestinal tissues (**Figures 6A, B**, **Table 1**). The percentage of CD4^-^CD8A^+^ T cells was comparable between the inflamed and noninflamed intestinal tissues in the same patients with CD. Interestingly, although the percentage of CD4^+^CD8A^-^ T cells in the noninflamed intestinal tissue was slightly less than in the inflamed intestine, the percentage of CD4^+^CD8A^+^ T cells in the noninflamed intestine was more than in the inflamed intestinal tissue (**Figure 6B**). Moreover, the percentage of CD4^-^CD8A^+^, CD4^+^CD8A^+^, CD4^+^CD8A^-^ T cells in TCRB^+^ cells did not differ between controls and noninflamed tissue of CD.

In the same patients with CD, the percentage of CD25^+^CD4^+^ cells among the total CD4^+^ T cells was increased in the inflamed compared with that in the noninflamed intestinal tissue as reported previously. Further, CD4^+^CD8A^+^ T cells among the total CD4^+^ T cells were reduced in the inflamed intestinal tissue compared to the noninflamed intestinal tissue (**Figures 6C, D**). The percentage of CD25^+^CD4^+^, CD4^+^CD8A^+^ T cells were comparable between controls and noninflamed tissue of CD. Taken together, these data suggest that the percentage of CD4^+^CD8A^+^ T cells is reduced in the inflamed tissue in CD patients.

We then stained for CD8B and CD27 to identify CD4^+^CD8A^+^ T cells based on the human scRNA-seq data (**Figure 4**). ScRNAseq data indicated CD8B expression was the featuring gene to identify hCD4^+^CD8A^+^ cluster, but few CD8B positive cells in both CD4^+^CD8A^–^ and CD4^+^CD8A^+^ T cell populations. Thus, the surface expression of CD8B is difficult to identify hCD4^+^CD8A^+^ cluster. Our previous data indicated CD27 expression of CD4^+^CD8A^+^ cells classified hTregs, thus CD27^–^CD4^+^CD8A^+^ cells were annotated into hMAIT and hCD4^+^CD8A^+^ cluster. The proportion of CD27^–^ cells in the noninflamed intestinal tissue was higher than in the inflamed intestinal tissue with CD4^+^CD8A^–^ and CD4^+^CD8A^+^ T cells (**Figures 6E, F**). Moreover, the proportion of CD27^–^CD8A^+^CD4^+^ cells among the TCRB^+^ T cells in noninflamed tissues was higher than in the inflamed tissue (**Figure 5G**). Taken together, the percentage of CD27^–^CD4^+^CD8A^+^ T cells annotated as hMAIT and hCD4^+^CD8A^+^ cluster is reduced in the inflamed intestine of patients with CD.

## Discussion

CD4-CTLs in humans are reported in patients who respond to the viral infection, hepatic virus, CMV, DENV, and COVID-19 vaccine. Recently CD4-CTLs have been detected in the blood of long-lived people; however, the existence of CD4-CTLs in the human gut has not been explored. In this study, we showed CTL genes, granzyme, perforin, and *NKG7* are expressed in hCD4^+^Treg, hCD4^+^MAIT, and hCD4^+^CD8A^+^ clusters, and CD4^+^ T cells in these three clusters expressed CD8A. We demonstrated that the hCD4^+^CD8A^+^ cluster is functionally close to mCD4-CTLs by integrating mouse and human scRNA-seq data. Our findings also showed that the proportion of CD4^+^CD8A^+^CD27^–^ T cells representing the hCD4CD8A and hMAIT clusters in the inflamed intestine of patients with CD is lower than that in the non-inflamed intestine of patients with CD.

CD4^+^CD8A^+^ T cells in the gut have been reported in previous studies (46). For instance, Christopher et al. demonstrated that IL-17A^+^CD8A^+^ cells were increased in active patients with UC (45). We did not detect IL-17A^+^CD8A^+^ cells but found IL-17A^+^CD4 T cells (conventional Th17 cells) in hTOX^+^ cluster. Cells in the hMAIT cluster expressed RORC, a transcriptional factor of IL-17A. Like in healthy donors, we did not detect the IL-17A^+^CD8A^+^ population in the intestinal tissue of patients with UC, which could be because the samples were not from the disease-active lesions in the small intestine of the patients. A recent study has identified small populations of CD4^+^CD8A^+^ cells in the cecum, small intestine, and mLN using scRNA-seq in various tissues, including small intestine and colon (44). However, the featuring genes of CD4^+^CD8A^+^ T cells were not identified in this study, as they grouped the population based on the CD4 and CD8A expression. Furthermore, a study reporting the scRNA-seq in patients with CD did not identify CD4^+^CD8A^+^ T cells (47). The study reported that part of the CD4^+^ T cells in cluster 0 expressed CD8A and GZMA. As CD4-CTLs in the hMAIT and CD4^+^CD8A^+^ clusters expressed CD4, CD8, and GZMA in our study, we inferred that the cluster 0 population identified in the previous study (47) may be similar to the hMAIT and CD4^+^CD8A^+^ clusters in this study.

Although the Treg populations in Corridoni et al.’s (46) and our studies were the same, the CD8+CD4+ cluster from Corridoni et al.’s dataset was not highly associated with hCD4^+^CD8A^+^ cluster from our dataset. The featuring genes of the CD8^+^CD4^+^ population in Corridoni et al.’s dataset were *CD4, CD8α, CCR6, IL7R, GZMA, ITGAE, KLRB1*, and NKG7, whereas in our study, the cells in the hCD4^+^CD8A^+^ cluster expressed all featuring genes listed. One reason for this difference may be the difference in the target cells; we targeted TCRB^+^ cells, whereas the previous study targeted CD8^+^ cells. Furthermore, they used colon biopsy specimens from patients with UC and healthy individuals, whereas we used surgical specimens from the small intestine. Nevertheless, further studies are required to confirm whether these two populations are the same or different.

In terms of cytokine production, CD4^+^CD8A^+^ T cells were reported to have a protective role and were found to produce IL-10 (22). Our data also support cells expressing IL10 were in the hTreg cluster, which belongs to CD4^+^CD8A^+^ T cells. CD4^+^CD8A^+^ cells are heterogeneous, and this heterogeneity may be the reason for the identification of multifunctional CD4^+^CD8A^+^ T cells in humans (22, 48).

To identify the human cluster whih corresponds with mCD4-CTLs, we used fast GSEA using mouse and human scRNA-seq data. mCD4-CTLs are in CD4^+^CD8A^+^ and CD4^+^IEL clusters (11, 12, 15, 17, 18). All mCD4-CTL population is highly correlated with hCD4^+^CD8A^+^ cluster. Moreover, we defined CD27 and CD8B as the marker to determine the hTreg, hMAIT, and hCD4^+^CD8A^+^ clusters. CD27, a T cell co-stimulatory molecule in both humans and mice (49) and is expressed on naïve and memory T cells (50). Cells in hTregs expressed CD27, whereas cells in hMAIT and hCD4^+^CD8A^+^ clusters did not express CD27. Cells in the hCD4^+^CD8A^+^ cluster expressed CD8B in human scRNA-seq, but we did not observe CD8B as surface protein. Therefore, further studies are required to confirm whether CD8B expression was reduced after antigen presentation or if they do not express as surface protein. Recently, the reinforced expression of CD8αβ^+^ on CD4^+^ T cells generated *in vitro* induced the expression of cytotoxic enzymes such as Gzmb and had an antitumor function (51–53). The results support that CD8αβ^+^ expression on CD4^+^ T cells is required to produce cytotoxic enzymes.

We did not observe the difference in the expression of cytotoxic genes in CD4^+^CD8A^+^ T cells between patients with IBD and controls, supporting the idea that cytotoxic gene expression in each cell was unaltered. Further study is needed as we did not compare the cytotoxic gene expression of the CD4-CTLs in inflamed tissue and those in noninflamed tissue of patients with IBD. Moreover, we cannot conclude whether the mCD4-CTLs and hCD4-CTLs are functionally equivalent population or not, as we claimed the similarity of these clusters depend on the gene expression. mCD4-CTLs have a suppressive function in murine intestinal inflammation. We revealed that CD27^–^CD4^+^CD8A^+^T cells were the majority of the CD4^+^CD8A^+^ T cells in the hCD4^+^CD8A^+^ cluster, and the percentage of the CD27^–^CD4^+^CD8A^+^ T cells among the total T cells in the intestines of patients with active CD was reduced compared with that in the intestines of patients with non-active CD. Cells in the hCD4^+^CD8A^+^ cluster may have a suppressive function like mCD4-CTLs.

In summary, we identified the human CD4-CTLs in the hTreg, hMAIT, and hCD4^+^CD8A^+^ clusters. By integrating mouse and human scRNA-seq datasets using fGSEA we show that the hCD4^+^CD8A^+^ cluster is similar to mCD4-CTLs. In addition, we highlight an approach for to identify human cell populations related to mouse cell populations. As it is difficult to delete target cells in humans, our approach may help determine the functional properties of several human cell populations.

## Data availability statement

The datasets presented in this study can be found in online repositories. The names of the repository/repositories and accession number(s) can be found below: https://ddbj.nig.ac.jp/resource/bioproject/PRJDB13408, PRJDB13408; https://www.ncbi.nlm.nih.gov/geo/, GSE210955.

## Ethics statement

The studies involving human participants were reviewed and approved by Keio University School of Medicine. The patients/participants provided their written informed consent to participate in this study. The animal study was reviewed and approved by Keio University School of Medicine.

## Author contributions

Conceptualization, ST, TS, C-CH, and JS. Methodology, KM, JM, YY, YA, IK, C-CH, and JS. Investigation, ST, TS, KM, YY, JM, YA, IK, YoH, AT, KeO, YuH, KT, and KoO. Visualization, TS, KM, JM, YA, IK, C-CH, and JS. Funding acquisition, TS, NH, HO, and TK. Project administration, TS. Supervision, TS, C-CH, JS, and TK. Writing – original draft, TS. Writing – review and editing, ST, KM, YY, TT, YM, NN, JS, and TK. All authors contributed to the article and approved the submitted version.

## Funding

Grants-in-Aid from the Japanese Society for the Promotion of Science (JSPS) (17K19668, 17H05082, 19K22624, 20H03665, 21K18272 to TS, 21K07084 to KO, 19K08402 to NH, 21H02905 to HO, and 20H00536 to TK). JST forest (TS). The Japan Agency for Medical Research and Development (19ek0109214 to TS and 21gm1510002h0001 to TK), The Keio University Medical Science Fund (Sakaguchi Memorial, Fukuzawa Memorial) (TS), The Takeda Science Foundation, the Mochida Memorial Foundation (TS), GSK Science Foundation (TS).

## Acknowledgments

We thank all the members in Department of Gastroenterology, Keio University.

## Conflict of interest

Author KM is an employee of Miyarisan Pharmaceutical Co. Ltd.

The remaining authors declare that the research was conducted in the absence of any commercial or financial relationships that could be construed as a potential conflict of interest.

## Publisher’s note

All claims expressed in this article are solely those of the authors and do not necessarily represent those of their affiliated organizations, or those of the publisher, the editors and the reviewers. Any product that may be evaluated in this article, or claim that may be made by its manufacturer, is not guaranteed or endorsed by the publisher.
